# CRISPR-*cas* system in the acquisition of virulence genes in dental-root canal and hospital-acquired isolates of *Enterococcus faecalis*

**DOI:** 10.1080/21505594.2020.1809329

**Published:** 2020-09-15

**Authors:** Pourya Gholizadeh, Mohammad Aghazadeh, Reza Ghotaslou, Mohammad Ahangarzadeh Rezaee, Tahereh Pirzadeh, Şükran Köse, Khudaverdi Ganbarov, Mehdi Yousefi, Hossein Samadi Kafil

**Affiliations:** a Drug Applied Research Center, Tabriz University of Medical Sciences, Tabriz, I.R. Iran; b Immunology Research Center, Tabriz University of Medical Sciences, Tabriz, I.R. Iran; c Department of Infectious Diseases and Clinical Microbiology, University of Health Sciences, Tepecik Training and Research Hospital, İzmir, Turkey; d Department of Microbiology, Baku State University, Baku, Azerbaijan; e Biotechnology Research Center, Tabriz University of Medical Sciences, Tabriz, I.R. Iran; f Stem Cell Research Center, Tabriz University of Medical Sciences, Tabriz, I.R. Iran

**Keywords:** *Enterococcus faecalis*, CRISPR-Cas system, virulence genes, phenotypic characteristics, hospital-acquired bacteria, dental-root canal bacteria

## Abstract

*Enterococcus faecalis* is one of the important causative agents of nosocomial and life-threatening infections in human. Several studies have demonstrated that the presence of CRISPR-*cas* is associated with antibiotic susceptibility and lack of virulence traits. In this study, we aimed to assess the phenotypic and genotypic virulence determinants in relation to CRISPR elements from the dental-root canals and hospital-acquired isolates of *E. faecalis*. Eighty-eight hospital-acquired and 73 dental-root canal isolates of *E. faecalis *were assessed in this study. Phenotypic screening of the isolates included biofilm formation, and gelatinase and hemolysis activities. Genotypical screening using PCR was further used to evaluate the presence of CRISPR elements and different virulence-associated genes such as *efaA, esp, cylA, hyl, gelE, ace, ebpR*, and *asa1*. Biofilm formation, gelatinase, and hemolysis activities were detected in 93.8%, 29.2%, and 19.2% of the isolates, respectively. The most prevalent virulence-associated gene was *ace*, which was followed by *efaA*, whereas *cylA* was the least identified. The presence of CRISPR1-*cas*, orphan CRISPR2, and CRISPR3-*cas* was determined in 13%, 55.3%, and 17.4% of the isolates, respectively. CRISPR elements were significantly more prevalent in the dental-root canal isolates. An inverse significant correlation was found between CRISPR-*cas* loci, *esp,* and *gelE*, while direct correlations were observed in the case of *cylA, hyl, gelE* (among CRISPR-loci 1 and 3), *asa1, ace*, biofilm formation, and hemolysis activity. Findings, therefore, indicate that CRISPR-*cas* might prevent the acquisition of some respective pathogenicity factors in some isolates, though not all; so selective forces could not influence pathogenic traits.

**Abbreviations:** BHI: brain-heart infusion agar; CRISPRs: Clustered regularly interspaced short palindromic repeats; Esp: Cell wall-associated protein; ENT: ear-nose-throat; ICU: intensive care units; OD: optical densities; PCR: polymerase chain reaction; SDS: sodium dodecyl sulfate; UTI: urinary tract infection

## Introduction

*Enterococcus faecalis*is a Gram-positive natural inhabitant of the mammalian digestive tract, including those of humans. It is also found in soil, plants, and dairy food products [1]. *E. faecalis*also behaves as an opportunistic pathogen causing life-threatening infections in humans, such as endocarditis, meningitis, septicemia, urinary tract infections, and others [2,3]. *E. faecalis* is one of the frequent isolates of the endodontic pathogens ranging in terms of prevalence from 30% to 90% of the cases [4,5]. The restriction system of *E. faecalis*enables the bacterium to acquire, accumulate, and further transfer genetic elements potentially encoding antibiotic resistance genes and virulence factors. These virulence factors include exoenzymes and adhesins. Cytolysin is encoded by *cyl* operon, which is carried by a plasmid or integrated into the chromosome, with both hemolysin and bacteriocin activity [6,7]. Gelatinaseis is encoded by the chromosomal *gelE* gene, which is a zinc metalloprotease; it can hydrolyze gelatin, fibrinogen, collagen, casein, and insulin [8]. Another secreted factor is hyaluronidase, which is encoded by the *hyl* gene [7]. *E. faecalis* endocarditis antigen is encoded by the*efaA*gene that affects pathogenicity [9]. Cell wall-associated protein (Esp), encoded by pheromone-responsive plasmids or the chromosomal *esp* gene, is involved in biofilm formation and immune evasion [9]. Aggregation substance, encoded by the*asa1* gene on the sex pheromone-plasmid pAD1, is a surface-bound glycoprotein which mediates the conjugative transfer of plasmids through the clumping of one *E. faecalis* to another and induces the formation of the cell-cell contact [10]. In addition, *ebpR*encodes an endocarditis- and biofilm-associated pilus regulator, which activates the *ebpABC* operon [11]. Another adhesion factor is a collagen-binding protein encoded by the *ace* gene, which mediates binding to collagen type I, collagen type IV, and laminin [12].

Clustered regularly interspaced short palindromic repeats (CRISPRs) loci and CRISPR-associated (Cas) protein-encoding genes are present in approximately 45% of eubacterial genomes sequenced [13–15]. There are three types of CRISPR loci in *E. faecalis* genome: CRISPR1-*cas*, orphan CRISPR2, and CRISPR3-*cas* [16–18]. CRISPR1-*cas* and orphan CRISPR2 were first found in the *E. faecalis* OG1RF strain: CRISPR1 is located between the OG1RF homolog of EF0672 and EF0673, which has the associated *cas* genes. CRISPR2 is located between the OG1RF homolog of EF2062 and EF2063, which is an orphan consisting only of spacers and palindromes, without any *cas* genes [16]. CRISPR3 was found in two genomes of the strains Fly1, as a fruit fly *E. faecalis*, and T11, as a urine *E. faecalis* isolate. CRISPR3 is located between the homologs of the *E. faecalis* V583 open reading frames EF1760 and EF1759 [18]. CRISPR1 possesses Nmeni subtype-specific genes *csn1* and *csn2* [16,18], while CRISPR3 only possesses *csn1*, not *csn2* [18]. Both CRISPR1 and CRISPR2 contain seven repeats of a 37 bp palindromic sequence with no homology to any sequences of the 29 bp spacer [16]. Nevertheless, due to small spacer sequences, it is likely that they are derived from the pheromone-responsive type plasmids, plasmids integrated within the *E. faecalis* V583 genome, and Enterococcal prophage and phage [18]. Recent studies have demonstrated that the CRISPR/Cas system has applications for genome engineering and exerts a strong selective pressure for the acquisition of virulence factors and antibiotic resistance in pathogenic bacteria [18–21]. Mojica et al., for instance, have suggested that the pathogenicity of bacteria is largely controlled by conjugative plasmids and bacteriophages on an evolutionary timescale. As well, those CRISPR spacers that target these mobile elements might affect bacterial pathogenicity and virulence traits [22].

In this study, we aimed to assess the phenotypic and genotypic virulence determinants in relation to CRISPR elements from the dental-root canals and hospital-acquired isolates of *E. faecalis*.

## Methods and materials

### Bacterial strains

This study was approved by the Regional Ethics Committee of Tabriz (Tabriz University of Medical Sciences, Tabriz, Iran, No. IR.TBZMED.REC.1397.188). A total of 88 isolates of *E. faecalis* were collected from EmamReza Teaching and Treatment Hospital and pediatric hospitals of Tabriz, Iran. The specimen sources of hospital-acquired isolates included urinary tract infection (UTI) (78, 88.6%), wound (7, 7.9%), and blood (3, 3.4%). The specimens were obtained from different wards including outpatients (35, 39.8%), intensive (23, 26.1%), intensive care units (ICU) (12, 13.6%), infectious ward (13, 14.8%), emergency ward (3, 3.4%), ear-nose-throat (ENT) (1, 1.1%), urology, and nephrology (1, 1.1%). Forty-two (47.7%) isolates were from male and 46 (52.3%) were from female cases. The age range of patients was from 2 months to 86 y, with a mean of 39.04 y. At the same time, in order to collect 73 dental-root canal isolates of *E. faecalis*, patients in need of endodontic treatment were referred to the clinic of the Faculty of Dentistry at Tabriz University of Medical Sciences, Tabriz, Iran. Forty-nine (67.1%) of the isolates were obtained from the males and 24 (32.9%) from the females. The age range of endodontic treatment patients was 12–66 y, with a mean of 32.41 y. Briefly to collect the isolates, after stages of access cavity preparation by the dentist, tooth, and its surroundings were washed by sterile saline solutions and disinfected with 30% hydrogen peroxide followed by 2.5% sodium hypochlorite. Root canal of teeth with no prior endodontic treatment and teeth with previous root canal treatment that showed secondary infection was removed by drill and endodontic K-files without using any chemical solvents. After sampling the single root canal and multi-root canal of the teeth, paper points were transferred to a tube containing Enterococcal broth (Becton Dickenson microbiology systems, Cockeysville, MD) and cultured on a bile esculin azide agar (Himedia, India) and incubated at 37°C for 24–48 h [4]. Suspected colony was identified by the standard procedures of microbiology [23,24] and genotype detection was performed by *ddlE* primer [25,26], as shown in Table 1. Both clinical and tooth identified isolates for further studies were stored in a trypticase soy broth containing 10% glycerol at −70°C.

**Table 1.** Primers used for the detection of virulence genes and CRISPR-associated genes.

Gene	Primer	Sequence (5ʹ–3ʹ)	PCR product length (bp)	References
*esp*	espF	GGAACGCCTTGGTATGCTAAC	95	[46]
	espR	GCCACTTTATCAGCCTGAACC		
*cylA*	cylF	ACTCGGGGATTGATAGGC	688	[47]
	cylR	GCTGCTAAAGCTGCGCTT		
*hyl*	hylF	ACAGAAGAGCTGCAGGAAATG	276	[11]
	hylR	GACTGACGTCCAAGTTTCCAA		
*efaA*	efaF	TGGGACAGACCCTCACGAATA	101	[48]
	efaR	CGCCTGTTTCTAAGTTCAAGCC		
*gelE*	gelF	TATGACAATGCTTTTTGGGAT	213	[47]
	gelR	AGATGCACCCGAAATAATATA		
*ace*	aceF	GGAGAGTCAAATCAAGTACGTTGGTT	101	[49]
	aceR	TGTTGACCACTTCCTTGTCGAT		
*ebpR*	ebpF	AAAAATGATTCGGCTCCAGAA	101	[11]
	ebpR	TGCCAGATTCGCTCTCAAAG		
*asa1*	asaF	GCACGCTATTACGAACTATGA	375	[47]
	asaR	TAAGAAAGAACATCACCACGA		
CRISPR1-*cas csn1*	For	CAGAAGACTATCAGTTGGTG	783	[18]
	Rev	CCTTCTAAATCTTCTTCATAG		
CRISPR1-*cas* loci	For	GCGATGTTAGCTGATACAAC	315	[18]
	Rev	CGAATATGCCTGTGGTGAAA		
CRISPR2 loci	For	CTGGCTCGCTGTTACAGCT	variable	[18]
	Rev	GCCAATGTTACAATATCAAACA		
CRISPR3-*cas csn1*	For	GCTGAATCTGTGAAGTTACTC	258	[18]
	Rev	CTGTTTTGTTCACCGTTGGAT		
CRISPR3-*cas* loci	For	GATCACTAGGTTCAGTTATTTC	224	[18]
	Rev	CATCGATTCATTATTCCTCCAA		

### Biofilm formation

Assessment of biofilm formation was done by quantitative biofilm formation in 96-well flat-bottom polystyrene microplates under static conditions for 48 h, as previously described [27,28]. Briefly, for each isolate, afresh colony cultured on a Muller-Hinton agar (Merck, Germany) containing 1% glucose was suspended in sterile saline and adjusted to 0.5 McFarland. Twenty microliters of the adjusted isolates was cultured in a 180-µl trypticase soy broth containing 1% glucose. After incubation for 48 h at 37°C, each well was washed by the 1X phosphate buffer saline (PBS; pH 7.4), fixed by methanol, and stained by 200 µl 0.1% crystal violet for 30 min at room temperature. The excess crystal violet was discarded and washed by water flow. Biofilm formation was measured by the absorbance of the supernatant after being solubilized in 33% acetic acid at 570 nm by using a microtiter plate reader (BioTeck, Winooski, USA). The biofilm formation of each isolate was tested in three independent 96-well microplates and the average of three optical densities (OD) was used as the final biofilm formation value. The cutoff absorbance for biofilm formation was considered higher than OD = 0.524, which was the absorbance of the biofilm produced by*E. faecalis*ATCC® 29,212™. The mean of the biofilm formation of each isolate was grouped based on their level of distribution (OD_570nm_ values) and categorized in quartiles higher than the cutoff absorbance and lower than the highest absorbance. Isolates whose absorbance of OD_570nm_ fell below 0.524 were classed as non-biofilm formation, while those with 0.525–1.087 and 1.088–1.650 were grouped as low and moderate biofilm formation, respectively. Isolates with a biofilm formation greater than 1.651 were also considered with high biofilm formation.

### Gelatinase production and hemolysis test

Hemolysis activity was assessed by blood agar plates prepared by brain–heart infusion agar (BHI, biomerieux, Poland, Ltd) containing 5% of the group ORh^+^ human blood. Cleared or green zone around the colonies was defined as hemolysis following incubation for 24 h at 37°C [29].

Production of gelatinase was assessed by the degradation of gelatin on the X-ray radiographic film, as described by Pickett et al. [30]. The heavy inoculum of individual isolates was cultured in the tubes containing 3 ml MHB and a strip of the X-ray radiographic film which had been cut into small strips (approximately 6 by 30 mm). The tubes were incubated for 24 h at 37°C and the cleared strip was defined as the production of gelatinase.

### Genotype detection of virulence and cas genes

Total DNA for each isolate was extracted by the tissue buffer boiling method. Briefly, 20 µl tissue buffer (0.25% sodium doedecyl sulfate (SDS) and 0.05 M NaOH) were mixed with one colony of bacterial isolate and incubated at 95°C for 10 min. The suspension was centrifuged at 13,000 *g* for 1 min, and 180 µl DNase free water was added. Genotype analysis for each isolate was accomplished based on the multiplex polymerase chain reaction (PCR) of virulence determinants encoding the cytolysin activator *cylA, hyl, esp, gelE, efaA, asa1, ace, ebpR*, CRISPR1-*cas*, CRISPR1-*cascsn1*, CRISPR2, CRISPR3-*cas,* and CRISPR3-*cascsn1*. Each of the primer sequences and the amplified size are shown in Table 1. Two microliters of total DNA was used for the multiplex PCR in a 25 µl reaction mixture. The mix for the detection of *esp*, cyl, *hyl* genes contained 12.5 µl of the PCR master mix (Yekta Tajhiz Azma, Iran), with 0.5 µM of each primer. The mix for ebp, *asa1,* and *efaA* had the same condition. The mix for the detection of *gelE* and *ace* contained 12.5 µl of the PCR master mix (Yekta Tajhiz Azma, Iran), 1.5 mM-additional MgCl_2_ and 0.5 µM of each primer. The mix for CRISPR1-*cascsn1*, CRISPR3-*cascsn1,* CRISPR1-*cas*, CRISPR3-*cas,* and CRISPR2 contained 12.5 µl of the PCR master mix (Yekta Tajhiz Azma, Iran), 1 mM additional MgCl2, and 10 mM of each primer. The amplification condition was carried out with the following thermal cycling conditions: initial denaturation at 95°C for 10 min, 34 cycles of amplification consisting of 95°C for 30 s, 30 s at 58°C for *esp, cylA, hyl*, 58°C for *efaA*, 56°C for gel, *ace*, 52°C for *ebpR, asa1*, 60°C for all *cas* genes, and 72°C for 45 s, with 72°C for 5 min in the final polymerization. PCR products were analyzed by electrophoresis in a 1% agarose gel at 100 V for 1 h in a 1X TBE buffer containing the DNA safe stain. The size of the PCR product was correlated with a 100 based-pair DNA ladder (YektaTajhizAzma, Iran) to confirm the conjunction with their expected PCR amplicon size. In addition, the PCR procedure for each isolate was carried out twice in the case of each primer in order to check the consistency and reproducibility.

### Statistical analysis

SPSS software, version 17.0 (Chicago, IL, USA) was used for statistical analysis. One-tailed Fisher’s exact test was used to compare the occurrence of CRISPR-*cas* loci in hospital-acquired and dental-root canal isolates and to evaluate the distribution of biofilm formation, gelatinase and hemolysin activities, and virulence genes among strains with CRISPR-*cas*. Student’s *t-*test was used to compare OD values among hospital-acquired and dental-root canal isolates. In addition, Spearman’s rank correlation was calculated between the presence of different virulence genes and CRISPR-*cas* loci among isolates. Significance was set at P ≤ 0.05.

## Results

**Figure 1.**
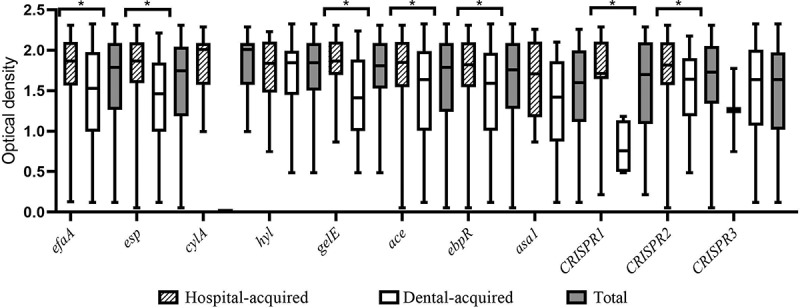
Biofilm formation absorbance by *E. faecalis* isolates according to the presence of virulence factors and CRISPR loci. (Error bars illustrate the minimum and maximum of three replicates of absorbance of the biofilm formation; *P-value was significant (*P*-value<0.05.)

**Figure 2.**
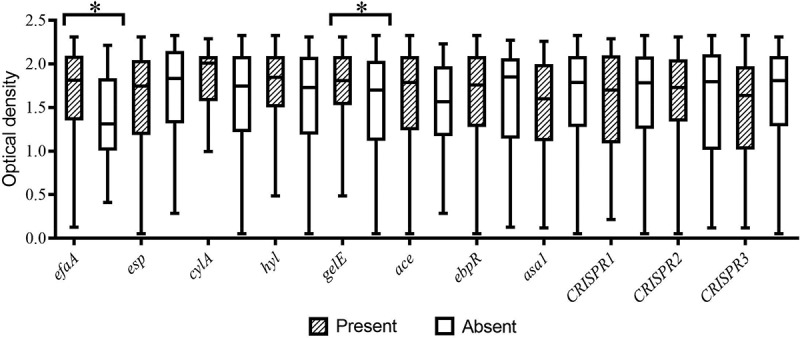
Biofilm formation absorbance association to virulence genes and CRISPR loci among *E. faecalis* isolates. (Error bars illustrate the minimum and maximum of three replicates of absorbance of the biofilm formation; *P-value was significant (*P*-value<0.05.)

**Figure 3.**
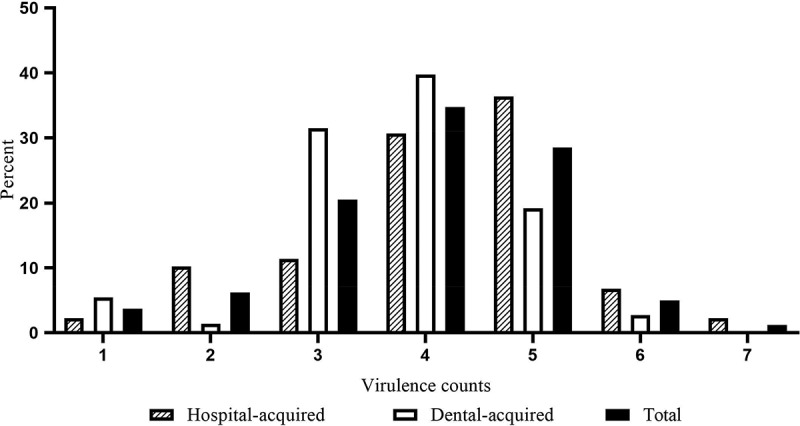
Distribution of virulence gene counts among *E. faecalis* isolates.

All isolates were investigated for the biofilm formation, in which the minimum, maximum, and average of biofilm formation (OD570 nm) were 0.054, 2.325, and 1.611, respectively. Most isolates showed strong biofilm formation (94, 58.4%), while 10 (6.2%) displayed no biofilm formation. Biofilm formation of hospital-acquired isolates was significantly higher than the dental-root canal isolates (*P* = 0.023). The biofilm formation absorbance according to the presence of virulence factors and CRISPR loci among *E. faecalis* isolates shown in Figure 1. Most of the isolates showed no gelatinase activity (70.8%), while hospital-acquired isolates significantly displayed the most gelatinase activity (*P* = 0.001). In addition, most isolates showed no hemolysis activity (80.7%), and all hemolysis activity was found in hospital-acquired isolates (19.2%). The most presence of the virulence genes among isolates were *ace* and*efaA*genes (88.8% and 85.1%, respectively), and the lowest one belonged to*cylA* and *asa1* (7.5% and 14.9%, respectively). The presence of *gelE* (contributing to gelatinase activity) and *cylA* (contributing to hemolysis activity) was significantly associated with phenotype gelatinase and hemolysis activity, respectively (*P* < 0.001, *P* = 0.013). In addition, the presence of *efaA, cylA*, and *gelE* was significantly more in hospital-acquired isolates, as compared to dental-root canal (*P* = 0.002, *P* < 0.001 and *P* = 0.008, respectively). Genotypic, and phenotypic determinants of hospital-acquired and dental-root canal isolates are shown in Table 2. The*efaA* and *gelE*harboring isolates had a higher biofilm formation than negative isolates in all isolates (*P* = 0.017 and *P* = 0.042, respectively). The biofilm formation absorbance association to virulence genes and CRISPR loci among *E. faecalis* isolates is shown in Figure 2. By comparing the presence of virulence genes among isolates, it was found that hospital-acquired isolates had higher virulence genes than dental-root canal isolates (*P* = 0.007), such that all isolates had at least one virulence gene. The distribution of virulence gene counts among *E. faecalis* isolates is presented in Figure 3. The number of virulence genes was 1–7 among hospital-acquired isolates and 16 in the case of dental-root canal isolates. Among hospital-acquired isolates, the presence of five and four virulence genes was the highest (36.4% and 30.7%, respectively); also, the presence of 4 and 3 virulence genes was the highest among isolates of the dental-root canal (39.7% and 31.5%, respectively).

**Table 2.** Genotypic and phenotypic determinants of hospital-acquired and dental-root canal isolates.

	*esp*	*cylA*	*hyl*	*efaA*	*gelE*	*ace*	*ebpR*	*asa1*	Gelatinase activity	Hemolysis			Biofilm formation			
Source	P (*n*)	P (*n*)	P (*n*)	P (*n*)	P (*n*)	P (*n*)	P (*n*)	P (*n*)	Positive (*n*)	*α*	β	γ	N	+	++	+++
**Hospital-acquired (88)**	73.9% (65)	13.6% (12)	15.9% (14)	93.2% (82)	37.5% (33)	88.6% (78)	79.5% (70)	15.9% (14)	39.8% (35)	29.5% (26)	5.7% (5)	64.8% (57)	4.5% (4)	6.8% (6)	20.5% (18)	68.2% (60)
UTIs (78)	78.2% (61)	15.4% (12)	14.1% (11)	93.6% (73)	34.6% (27)	89.7% (70)	78.2% (61)	16.7% (13)	39.7% (31)	29.5% (23)	6.4% (5)	64.1% (50)	5.1% (4)	3.8% (3)	21.8% (17)	69.2% (54)
Non-UTIs (10)	40% (4)	0	30% (3)	90% (9)	60% (6)	80% (8)	90% (9)	10% (1)	40% (4)	30% (3)	0	70% (7)	0	30% (3)	10% (1)	60% (6)
**Dental root (73)**	68.5% (55)	0	24.7% (18)	75.3% (55)	19.2% (14)	89% (65)	84.9% (62)	13.7% (10)	16.4% (12)	0	0	100% (73)	8.2% (6)	19.2% (14)	26% (19)	46.6% (34)
*P*-value^§^	0.282	<0.001	0.118	0.002	0.008	0.570	0.249	0.435	0.001	<0.001			0.023			
**Total (161)**	71.4% (115)	7.5% (12)	19.9% (32)	85.1% (137)	29.2% (47)	88.8% (143)	82% (132)	14.9% (24)	29.2% (47)	16.1% (26)	3.1% (5)	80.7% (130)	6.2% (10)	12.4% (20)	23% (37)	58.4% (94)

UTIs, urinary tract infections; non-UTIs: other isolates containing wound and blood; P: presence; N: negative; α: alpha hemolysin; β: beta hemolysin; γ: none hemolysin; +: low; ++: moderate; +++: strong

§ One-tailed Fisher’s exact test was used for comparison of hospital-acquired and dental-root canal groups.

**Figure 4.**
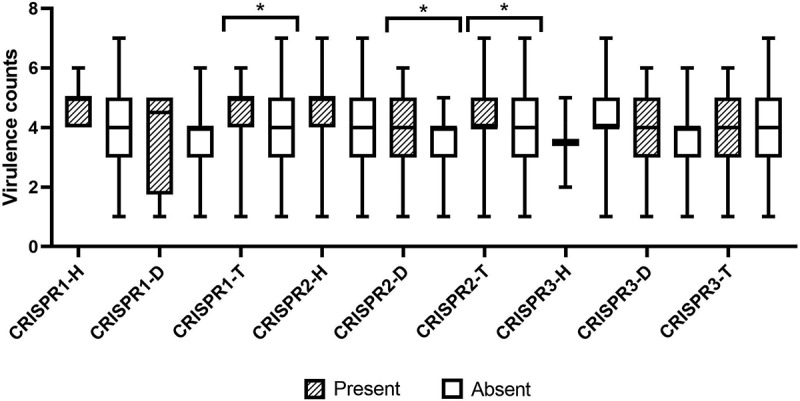
Virulence gene counts association to CRISPR loci among *E. faecalis* isolates. (Error bars illustrate the minimum and maximum of virulence gene counts; *P-value was significant (*P*-value <0.05; H: Hospital-acquired; D: Dental-acquired; T: Total).

The occurrence of CRISPR-*cas* is shown in Table 3. Overall, the presence of CRISPR1-*cas* loci in dental-root canal isolates (4 of 73) was lower than that of hospital-acquired isolates (17 of 88) (*P* = 0.008), whereas the presence of CRISPR3-*cas* in dental-root canal isolates (26 of 73) was higher than that of hospital-acquired isolates (2 of 88) (*P* < 0.001); also, orphan CRISPR2 made no difference between hospital-acquired and dental-root canal isolates. None of the isolates had, however, both of CRISPR1-*cas* and CRISPR3-*cas*, as well as CRISPR1-*cas*, orphan CRISPR2, and CRISPR3-*cas*, at the same time. The isolates were more likely to harbor orphan CRISPR2 than CRISPR1-*cas* and CRISPR3-*cas*.In addition, the presence of orphan CRISPR2 was significantly correlated with CRISPR1-*cas* (*P* = 0.031, correlation coefficient = 0.163), whereas it was not significant with CRISPR3-*cas*. At least one CRISPR-*cas* locus was found in 106 (65.8%) of all isolates. The results, therefore, showed the isolates containing high virulence genes tended to have more frequently investigated *cas* genes. The presence of CRISPR1 and CRISPR 2 was significantly correlated with high distribution of virulence gene numbers (*P* = 0.010 and *P* = 0.011, respectively). The virulence gene counts association to CRISPR loci among *E. faecalis* isolates is shown in Figure 4. Overall, the absence of CRISPR1-*cas*and one of CRISPR1 or CRISPR3weresignificantly correlated with the absence of the *esp* gene (*P* = 0.005, correlation coefficient = 0.204 and*P* = 0.033, correlation coefficient = 0.157, respectively). In addition, the presence of either CRISPR1-*cas* or orphan CRISPR2 and either CRISPR3-*cas* or orphan CRISPR2 was significantly correlated with the presence of *ace* and the absence of *gelE*, respectively (*P = 0*.019, correlation coefficient = 0.185 and *P = *0.014, correlation coefficient = 0.184, respectively). In addition, presence of CRISPR1-*cas* was significantly correlated with the absence of *hyl* (P = 0.048, correlation coefficient = −0.147). Other significant correlations were found between the absence of CRISPR1 and the absence of *cylA*and*asa1* (*P* < 0.05, correlation coefficient = 0.171 and 0.149, respectively), and between the absence of CRISPR2 and the absence of *gelE* (*P =* 0.001, correlation coefficient = 0.248). In hospital-acquired isolates, a significant correlation was found between the absence of CRISPR loci and the absence of *gelE, asa1*, gelatinase and hemolysis activity (*P* < 0.05); in dental-root canal isolates, a significant correlation was found between the absence of CRISPR3-*cas* and the absence of gelatinase (*P* = 0.003, correlation coefficient = 0.365), between the absence of either CRISPR1-*cas* or CRISPR2-*cas* and the absence of *gelE* (*P* = 0.021, correlation coefficient = 0.265), and between the presence of orphan CRISPR2 and either orphan CRISPR2 or CRISPR3-*cas* and biofilm production (*P* = 0.046, correlation coefficient = 0.247 and*P* = 0.044, correlation coefficient = 0.263, respectively) (see Table 4).

**Table 3.** The presence of CRISPR-*cas* type in hospital-acquired and dental-root isolates of *E. faecalis.*

CRISPR	CRISPR1-cas	CRISPR2	CRISPR3-cas	CRISPR1-cas or CRISPR2	CRISPR1-cas or CRISPR3-cas	CRISPR2 or CRISPR3-cas	CRISPR1-cas and CRISPR2	CRISPR1-cas and CRISPR3-cas	CRISPR2 and CRISPR3-cas	CRISPR1-cas and CRISPR2 and CRISPR3-cas
Hospital-acquired isolates (88)	19.3% (17)	53.4% (47)	2.3% (2)	59.1% (52)	21.6% (19)	54.5% (48)	13.6% (12)	0	1.1% (1)	0
Dental root isolates (73)	5.5% (4)	57.5% (42)	35.6% (26)	58.9% (43)	42.5% (31)	72.6% (53)	5.5% (4)	0	20.5% (15)	0
*P*-value^§^	0.008	0.358	<0.001	0.554	0.004	0.014	0.070	NS	<0.001	NS
Total (161)	13% (21)	55.3% (89)	17.4% (28)	59% (95)	31.1% (50)	62.7% (101)	9.9% (16)	0	9.9% (16)	0

NS, not significant.

§ One-tailed Fisher’s exact test was used for comparison of hospital-acquired and dental-root canal groups.

**Table 4.** Association between genotypic and phenotypic characteristics and the occurrence of CRISPR-*cas* in *E. faecalis.*

Gene	CRISPR1-present	CRISPR1-absent	*P*-value	CRISPR2-present	CRISPR2-absent	*P*-value	CRISPR3-present	CRISPR3-absent	*P*-value	CRISPR1 or CRISPR2-present	CRISPR1 or CRISPR2-absent	*P*-value	CRISPR1 or CRISPR3-present	CRISPR1 or CRISPR3-absent	*P*-value	CRISPR2 or CRISPR3-present	CRISPR2 or CRISPR3-absent	*P*-value
*esp*-present	20	95	0.005	64	51	0.509	21	94	0.417	68	47	0.549	41	74	0.033	72	43	0.554
*esp*-absent	1	45		25	21		7	39		27	19		9	37		29	17	
*cylA*-present	4	8	0.053	6	6	0.464	0	12	0.092	7	5	0.594	4	8	0.544	6	6	0.258
*cylA*-absent	17	132		83	66		28	121		88	61		46	103		95	54	
*hyl*-present	1	31	0.048	20	12	0.237	6	26	0.500	21	11	0.260	7	25	0.149	23	9	0.161
*hyl*-absent	20	109		69	60		22	107		74	55		43	86		78	51	
*efaA*-present	20	117	0.139	75	62	0.461	22	115	0.214	81	56	0.557	43	94	0.518	86	51	0.575
*efaA*-absent	1	23		14	10		6	18		14	10		7	17		15	9	
*gelE*-present	7	40	0.415	35	12	0.001	5	42	0.108	36	11	0.003	12	35	0.217	36	11	0.014
*gelE*-absent	14	100		54	60		23	91		59	55		38	76		65	49	
*ace*-present	20	123	0.282	84	59	0.012	26	117	0.358	89	54	0.019	46	97	0.285	94	49	0.027
*ace*-absent	1	17		5	13		2	16		6	12		4	14		7	11	
*ebpR*-present	18	114	0.451	75	57	0.263	24	108	0.399	80	52	0.250	42	90	0.418	85	47	0.235
ebpR-absent	3	26		14	15		4	25		15	14		8	21		16	13	
*asa1*-present	6	18	0.067	15	9	0.293	3	21	0.363	17	7	0.146	9	15	0.303	16	8	0.425
*asa1*-absent	15	122		74	63		25	112		78	59		41	96		85	52	
Biofilm-positive	19	132	0.384	86	65	0.092	27	124	0.453	91	60	0.176	46	105	0.377	97	54	0.117
Biofilm-negative	2	8		3	7		1	9		4	6		4	6		4	6	
Gelatinase-positive	4	43	0.203	29	18	0.190	10	37	0.268	30	17	0.268	14	33	0.490	32	15	0.236
Gelatinase-negative	17	97		60	54		18	96		65	49		36	78		69	45	
Hemolysis-positive	4	27	0.623	19	12	0.293	0	31	0.001	19	12	0.469	4	27	0.010	19	12	0.504
Hemolysis-negative	17	113		70	60		28	102		76	54		46	84		82	48

## Discussion

In this study, we determined the occurrence of CRISPR loci and the content of virulence factors in *E. faecalis* strains isolated from different infectious sources as a pathogenic organism and the dental-root canal of patients. We found that the presence of CRISPR1 and CRISPR3 loci was varied among *E. faecalis* strains. The abundance of CRISPR1 among the dental-root canal isolates was significantly lower than that of hospital-acquired ones, whereas the reverse was significantly true for CRISPR3. These results were consistent with those obtained by Burley et al. study [31], who found the presence of CRISPR3-*cas*was significantly more in endodontic strains, as compared to hospital-acquired strains, and the majority of strains had CRISPR3. While these results were interesting, the reasons were not clear. In addition, we found that the presence of orphan CRISPR2 was more among *E. faecalis* strains in comparison to CRISPR1-*cas* and CRISPR3-*cas*, while CRISPR2 Lacks of *cas* genes. Palmer et al. [18] and Hullahalli et al. [32]suggested that CRISPR2 is functional for sequence interference and is functionally linked to CRISPR1-Cas or CRISPR3-Cas.

The results revealed that the presence of CRISPR loci was not significantly associated with a less number of virulence factors. There are several virulence factors in *E. faecalis* which play such roles as antiphagocytosis, adherence, biofilm formation, exoenzyme, toxin, and quorum sensing system. Although several studies have reported that there is no clear relation between origin isolation or a single gene and pathogenicity, and perhaps the surface proteins of *E. faecalis*cannot be considered as virulence factors [9,33,34], we found a correlation between the absence of CRISPR1-*cas* and the absence of the*esp* gene (*P*-value = 0.009, coefficient correlation = 0.204) and a correlation between the absence of CRISPR1-*cas* and the absence of *cylA* (*P*-value = 0.03, coefficient correlation = 0.171) and *asa1* (*P*-value = 0.06, coefficient correlation = 0.149) genes. In addition, there was a correlation between the absence of single or multi-CRIPSR loci and the absence of some virulence factors. The cytolysin operon, *cob* and *esp* genes reside in the same pathogenicity island, which are located on either the chromosome or on large pheromone-responsive plasmids such as pAD1 [35,36]. The *esp* gene encodes a large surface protein with a variable number of highly conserved 82 amino acids repeats, contributing to the promotion of primary attachment, colonization and biofilm formation of*E. faecalis* [36]. Our results, therefore, showed that the presence of*efaA,esp, gelE, ace,* and *ebpR* genes were significantly associated with biofilm formation among the hospital-acquired isolates and *efaA* and *gelE* genes were significantly associated with biofilm formation in all *E. faecalis* isolates. Conflict outcomes have been, however, published regarding the role of the genes of biofilm formation. Duggan et al., for example, suggested that *asa1, cylA, esp* and *gelE*were not associated with biofilm formation in the oral and endodontic isolates of *E. faecalis* [37], which is compatible with our results. In addition, the results revealed that 13.6% of hospital-acquired isolates carried the*cylA* gene, but only 35.2% of the isolates expressed hemolysin activity (both alpha and beta hemolysis). Several studies such as Sun et al. [38], Sedgley et al. [39] and Lindenstrauß et al. [40] have also determined 38%, 36% and 33.3% of the chronic periodontitis, endodontic, and clinical and food isolates of *E. faecalis* to be capable of producing hemolysis, respectively. These differences may be due to the differences in the types of blood used for the determination of the hemolysis activity, while we used human blood, others have employed horse and sheep blood. In addition, Sun et al. [38] and Sedgley et al. [39] reported the distribution of the *cylA* gene was detected only in 17% and 18.18% of the isolates, respectively; this was compatible with our results. These results may be due to such environmental factors as *in vitro* and *in vivo* conditions used to test for phenotypic characters, which could strongly influence gene expression [41] and can be the cause of the differences between our results and those obtained by others in the case of hemolysis activity. In addition, hemolysin activity was encoded by *cyl* operon in *E. faecalis*, where *cylA* is the only reading frame required for the expression of component A, a serine protease. As well, there is no association between CRISPR1-*cas*, biofilm-formation, and hemolysis activity. Several studies have reported that CRISPR loci play an inverse role in some virulence factors and acquisition of antibiotic resistance [18,31,40], such as Palmer and Gilmore’s study [42] and Burley et al.’s study [31], reporting that CRISPR loci were inversely associated with antibiotic resistance and some virulence factors in *E. faecalis* strains. In addition, similar to our results, Toro et al. [43] and Touchon et al. [44] reported that there was no significant association with CRISPR-*cas* and acquisition of integrons, plasmids, antibiotic resistance and virulence genes in *Escherichia coli*. However, an analysis of 370 other Archaeal and Eubacteria genomes showed that there was potential evidence for the propagation of CRISPR-*cas* genes to occur via horizontal gene transfer [45]. These findings, therefore, suggested that CRISPR loci could potentially inhibit or prevent some or part of the virulence factors and Pathogenicity Island could not serve as the selective forces to influence the pathogenic traits of *E. faecalis*.

## Conclusion

The findings of this study indicated that CRISPR-*cas*mightprevent the acquisition of some respective pathogenicity factors in some isolates, though not all; significant inverse correlations were found between CRISPR-*cas* loci, *esp* and *gelE*, while direct ones were found in *cylA, hyl, gelE* (between some CRISPR-loci), *asa1, ace*, biofilm formation, gelatinase, and hemolysis activities. However, other studies demonstrated that CRISPR-*cas* could prevent the acquisition of antibiotic resistance genes in *E. faecalis* and other bacteria. Further studies can determine the exact role of CRISPR-*cas* in the pathogenesis of Enterococcal infections.

## Data Availability

The datasets used in this study are available from the corresponding author on reasonable request.
